# Trial Forge Guidance 4: a guideline for reporting the results of randomised Studies Within A Trial (SWATs)

**DOI:** 10.1186/s13063-024-08004-0

**Published:** 2024-03-12

**Authors:** C. E. Arundel, L. K. Clark, A. Parker, D. Beard, E. Coleman, C. Cooper, D. Devane, S. Eldridge, S. Galvin, K. Gillies, C. E. Hewitt, C. Sutton, D. J. Torgerson, S. Treweek, the PROMETHEUS GROUP

**Affiliations:** 1 York Trials Unit, Department of Health Sciences, Faculty of Science, Lower Ground Floor ARRC Building, University of York, York, YO10 5DD UK; 2 Nuffield Department of Orthopaedics, Rheumatology and Musculoskeletal Sciences, Botnar Research Centre, University of Oxford, Headington, Oxford UK; 3 Clinical Trials Research Unit, University of Sheffield, Sheffield, UK; 4 HRB-Trials Methodology Research Network, University of Galway, Galway, Ireland; 5 School of Nursing and Midwifery, University of Galway, Galway, Ireland; 6 Evidence Synthesis Ireland and Cochrane Ireland, University of Galway, Galway, Ireland; 7 Wolfson Institute of Population Health, Queen Mary University of London, London, UK; 8 Health Services Research Unit, University of Aberdeen, Aberdeen, UK; 9 School of Health Sciences, The University of Manchester, Manchester, UK

**Keywords:** Study within A Trial, SWAT, Embedded randomised controlled trial, Reporting guideline, Reporting standard

## Abstract

**Background:**

Evidence to support decisions on trial processes is minimal. One way to generate this evidence is to use a Study Within A Trial (SWAT) to test trial processes or explore methodological uncertainties. SWAT evidence relies on replication to ensure sufficient power and broad applicability of findings. Prompt reporting is therefore essential; however, SWAT publications are often the first to be abandoned in the face of other time pressures. Reporting guidance for embedded methodology trials does exist but is not widely used. We sought therefore to build on these guidelines to develop a straightforward, concise reporting standard, which remains adherent to the CONSORT guideline.

**Methods:**

An iterative process was used to develop the guideline. This included initial meetings with key stakeholders, development of an initial guideline, pilot testing of draft guidelines, further iteration and pilot testing, and finalisation of the guideline.

**Results:**

We developed a reporting guideline applicable to randomised SWATs, including replications of previous evaluations. The guideline follows the Consolidated Standards for Reporting Trials (CONSORT) statement and provides example text to ensure ease and clarity of reporting across all domains.

**Conclusions:**

The SWAT reporting guideline will aid authors, reviewers, and journal editors to produce and review clear, structured reports of randomised SWATs, whilst also adhering to the CONSORT guideline.

**Trial registration:**

EQUATOR Network – Guidelines Under Development (https://www.equator-network.org/library/reporting-guidelines-under-development/reporting-guidelines-under-development-for-clinical-trials/#SWAT). Registered on 25 March 2021.

## Background

There is a significant amount of avoidable waste in producing and reporting evidence from randomised trials [1]. Some of this waste stems from uncertainty about how best to undertake specific trial processes: recruitment and retention of trial participants, for instance, are essential to nearly all trials but remain a persistent challenge [2, 3]. Despite this, the evidence available to support trialists’ decisions about recruitment and retention is minimal [4, 5]. Evidence on how best to undertake other trial processes will likely be even worse.

One way to generate trial process evidence is to embed a Study Within A Trial (SWAT) within a host trial to test trial process alternatives (e.g. different trial retention or data collection strategies) or explore why processes are undertaken as they are (e.g. exploration of reasons for non-consent) [6, 7]. SWATs may be randomised or non-randomised depending on the question being asked and may be completed in a single-host trial or across multiple-host trials. Evaluations in multiple host trials can either be done at the same time or individually over an extended period. A randomised evaluation of a research process may also be embedded within other research designs, e.g. within a prospective cohort (Trial Within A Cohort TWIC).

Most SWATs to date have focused on recruitment and retention strategies. The number of such SWATs is increasing, with 45 recruitment studies identified in a 2010 systematic review and 68 in the 2018 update of that review [4]. For retention, there were 38 studies identified in 2014 and 72 by 2020 [5]. There have been fewer SWATs in other trial process areas and so further advances would be welcome.

A central driver for the increase in SWAT activity, especially in recruitment and retention, is the promotion of SWATs through funded programmes such as MRC-Start [8], initiatives such as Trial Forge [9] and the Health Research Board—Trials Methodology Research Network (Ireland), and the availability of dedicated SWAT funding from funders such as the UK National Institute for Health and Care Research (NIHR) [10], the Health Research Board in Ireland [11] and Accelerating Clinical Trials (Canada) [12]. The PROMoting the USE of SWATs (PROMETHEUS) research programme, a programme of coordinated recruitment and retention SWATs, has added further to this by overseeing 42 SWATs in 31 trials [13].

The need for prompt and transparent reporting of research findings is well known. SWAT evidence depends on replication to ensure sufficient participants are involved and to support broad applicability by including contextual variation across a wide range of trials with different clinical populations. For those replications to improve trial process decisions, SWATs need to be published and reported. However, discussions with SWAT researchers suggest that SWATs are often one of the first publications to be abandoned in the face of time pressures. More empirical evidence from the Cochrane reviews on recruitment and retention [4, 5] shows that even basic information for risk of bias assessment is poorly reported in 48% of the included SWATs (i.e. risk of bias was assessed as unclear).

Reporting guidance for the reporting of embedded recruitment trials does exist [14] but is not widely used, perhaps because it seems too demanding for what is often a small study nested within a large trial. As part of the PROMETHEUS Programme, we sought to build on these guidelines to develop a more straightforward standard, which still adheres to the CONSORT guideline [15] but is more focused on consistent, concise, and rapid reporting of SWATs. Like the original guidance, our guideline is tailored to reporting randomised SWATs.

### Scope of the guideline

Given that clinical trial evidence informs healthcare decision-making, it follows that evidence from SWATs has the potential to improve decision-making in trial processes. However, to realise this potential, we need to remove the barriers to effective reporting of SWATs. The use of a SWAT reporting guideline can help us to achieve this goal.

This SWAT reporting guideline was developed to aid authors in producing clear, structured reports of randomised SWATs conducted in host trials done both separately and simultaneously. Moreover, this guideline also provides a useful tool for reviewers and journal editors.

### SWAT reporting guideline rationale

Development of the guideline was initiated because of several common problems identified through the PROMETHEUS programme [13, 16, 17]. Discussion with members of the Trial Forge SWAT Network also identified more straightforward publication of SWATs as an important, medium-term priority [18]. Common problems reported by SWAT researchers concerning the publication of SWATs included:

- A lack of time to write a SWAT publication. This concern stemmed from researchers assuming a SWAT publication needs to be a lengthy document like that for the host trial(s) in which the SWAT was embedded.
- The SWAT publication is not considered a priority compared to the main host trial publication(s).
- A lack of confidence and knowledge about how to generate and submit a SWAT publication.
- A lack of SWAT-focused journals and/or a reluctance from other non-methodological journals to publish such work.
- A lack of funding to support SWAT publications in peer-reviewed open-access journals.
- Reviewer feedback that reflects a misunderstanding of SWAT methodology.

### Development of the SWAT reporting guideline

The PROMETHEUS programme faced challenges implementing the earlier guidance [14], which led its Programme Management group to propose a new reporting format in 2019. The goal was to make publishing SWATs easier by developing a concise reporting guideline of 1000 words or less. This new format would be simpler to write, and potentially more cost-effective, as shorter articles often have lower open-access publication charges.

A further meeting was convened in July 2019 to discuss this proposal more widely with PROMETHEUS Programme Management team members, authors of previous guidelines for reporting embedded trials [14], and a representative from the BMC journal *Trials*. Meeting participants were provided with example publications (one was in development for peer-reviewed submission [19], and the other was reworked from a previously published SWAT [20]), written in under 1000 words for review and consideration. It was agreed by consensus that the methodological information included was sufficiently robust for reporting the SWATs (i.e. in line with CONSORT) and would enable inclusion of the results into an aggregate meta-analysis.

Following this, a further meeting was convened with the authors of previous guidelines for reporting embedded trials [14] to discuss the proposed guideline. The consensus was that the proposed guideline should be developed to build on knowledge derived from the PROMETHEUS programme. Suggested additional revisions included the inclusion of the term ‘SWAT’ as opposed to ‘embedded trial’ and ensuring that any developed guideline remained CONSORT compliant [15].

The PROMETHEUS Programme Management team developed a draft guideline for concise SWAT reporting, which was then reviewed and refined by the wider team. At this stage, the team conceded that a word count of 1000 words was too ambitious and arbitrary, making it challenging to include sufficient details of the host trial(s) and report on complex interventions and designs. Therefore, we dropped the word limit to allow for more comprehensive reporting, if needed. The need for an initial meta-analysis if the reported SWAT was the second replication or updated meta-analysis (for replications after that) was also added to ensure that the accumulated effect of the intervention was reported.

The guideline was then circulated to a wider stakeholder group for comment. This group included five national and one international trial methodologist, affiliated with academic institutions (*n* = 5), and one methodologist working for a commercial contract research organisation. The guideline was also reviewed by a patient and public involvement (PPI) contributor. The trial methodologist stakeholder group suggested that the best way to assist researchers in writing and publishing their SWAT would be to provide a reporting template that included exemplary wording for each of the guideline’s sections. The PPI member recommended that technical language throughout be simplified. The guideline was updated accordingly using a CONSORT-style tabulation, which included exemplary wording, with attempts made to simplify language where possible.

Revisions were also made to the exemplar text for randomisation and allocation concealment after it was identified in an updated Cochrane review of strategies for improving retention to RCTs that many SWATs had moderate or low-grade certainty evidence due to poor reporting of these items [5]. The Cochrane review found that out of 68 studies, 42 (62%) inadequately reported allocation concealment and 28 (41%) inadequately reported sequence generation [5]. Minor changes to the guideline also included encouraging the use of standard keywords in SWAT reporting, which can help users and systematic reviewers find relevant SWATs through electronic searches.

### Pilot testing of the SWAT reporting guideline

Throughout the review and development process, we continued to assess the iterations of the guideline by asking colleagues at the York Trials Unit, University of York, and PROMETHEUS Programme team members and collaborators to use the most current version of the guideline when writing up a SWAT for publication [21–25]. The *Research Methods in Medicine and Health Sciences* journal also provided a version of the guideline to support their SWAT special issue in September 2022 [26].

The final draft guidelines were then tested in two further SWAT publications (one recruitment SWAT, one retention SWAT) to identify any necessary further edits. Some minor clarifications were made to the exemplar text and instances of duplication removed to streamline the guideline. References to PROGRESS-PLUS criteria were also added to ensure sufficient reporting of equality, diversity, and inclusion aspects [27].

For transparency, the development of this reporting guideline was registered with the EQUATOR network on 25 March 2021.

### SWAT reporting guideline

The final SWAT reporting guideline is given in Table 1 and applies to all reports of randomised SWAT evaluations, including replications of previous evaluations. For replication SWATs, it is recommended to include a cumulative meta-analysis of all replications to date in the publication, if feasible. For coordinated simultaneous SWATs (e.g. conducted across multiple host trials at the same time), the report should summarise all included host trials and combine the results in a cumulative meta-analysis.

The guideline shown in Table 1 is composed of 40 individual components. The vast majority of the components (*n* = 35, 87.5%) correspond to items in the CONSORT checklist of 2010 [15] and were selected by Madurasinghe et al. for inclusion in their guidance for reporting embedded recruitment studies [14]. Each of these 35 items has been reviewed and guidance and suggested text provided to accurately reflect the conduct of, and guide researchers in the reporting of specific nuances relevant to, SWAT design, delivery, and reporting. Of the remaining five items, four were new items: Keywords—Item 1c; Presentation of binary outcomes—Item 17b; Costs of the SWAT—Item 17c; and Implications for practice and trials research—Item 22, and one item was a modification of an existing CONSORT 2010 checklist item (Discussion) which was amended to reorder the section structure to improve reporting flow.

**Table 1:** The SWAT reporting guideline

**CONSORT 2010 item to be included in publication **[15]		**Additional information and example text shown in italics where possible**
**Title and Abstract**		
**1a**	The term ‘SWAT’ should be used in the title	The SWAT registry number should be included if available: *SWAT [insert number]: [insert title of SWAT]*
**1b**	Structured summary	Structured using these headings: Background, Methods, Results, Conclusion Details of the host trial(s) included in which the SWAT intervention was evaluated
**1c**	Keywords	Include: ‘SWAT’; ‘Study Within A Trial’; the trial process targeted (e.g. ‘recruitment methods’); embedded randomised controlled trial
**Introduction; Background and objectives**		
**2a**	Scientific background and explanation of rationale for the SWAT	Justify the need for the SWAT; cite systematic review evidence where appropriate Replication SWAT: Also cite previous SWAT evaluations undertaken as part of the rationale
**2b**	Specific objectives or hypotheses for the SWAT	State SWAT question as objective *Does [insert SWAT intervention] increase/decrease [outcome] compared to [comparator] in [participants]?*
**Methods; Trial design**		
**3a**	Description of the SWAT (such as parallel, factorial, cluster), including allocation ratio	Describe the trial design and allocation ratio: *A [insert number of trial arms and trial design] SWAT was undertaken with an allocation ratio of [insert allocation ratio] (intervention detail vs control detail)* State where the SWAT protocol is registered: *The SWAT protocol (number) can be found at [insert details of SWAT repository link]* If SWAT protocol is not registered, include it as an appendix Host trial(s): *The SWAT was embedded in the [insert host trial name(s)]* Reference the host trial’s registration number(s) and if the protocol(s) for the host trial is/are available elsewhere or include a link to the study project page(s) Provide a brief description of the host trial(s) using PICO format. At a minimum, age, gender, and ethnicity should be reported per group in addition to any demographics deemed relevant by the host trial team(s); however, we encourage authors to refer to and report in accordance with PROGRESS-PLUS [27] where feasible. If the SWAT was conducted across multiple host trials at the same time, a description of each host trial should be provided *Host trial Participants; Intervention; Comparator; Outcomes* State the ethical approval arrangements for the SWAT: *The SWAT was approved by the Research Ethics Committee [insert name/reference number]* If changes to the SWAT occurred: *The following changes occurred once the SWAT started [insert text]*
**3b**	State changes (with reasons) to methods of SWAT following commencement	
**Participants**		
**4a**	State eligibility criteria in SWAT, including differences to those from the host trial(s)	State participant eligibility. This can be tabulated
**4b**	Include setting(s) and location(s) where SWAT data was collected	Describe SWAT data collection methods: *SWAT data were collected in the following settings/locations [insert text] using the following methods [e.g. face to face, postal follow-up, telephone follow-up, electronic data collection]*
**Interventions**		
**5**	Describe SWAT intervention to enable replication, including how and when interventions were administered and recruitment dates	Briefly describe the SWAT intervention and control. Reference to the SWAT protocol for further details is acceptable if the protocol is available to the reader
**Outcomes**		
**6a**	State primary and secondary outcome measures for the SWAT Include how and when they were assessed	State the primary and outcome measures for the SWAT: *Primary outcome measure: [insert information including how/when/who assessed]* *Secondary outcome measure(s): [insert information including how/when/who assessed]* This information can be tabulated If appropriate: *The following changes occurred once the SWAT started [insert text]*
**6b**	Include changes (and reasons) to SWAT outcomes after commencement	
**Sample size**		
**7a**	How sample size was determined for the SWAT	SWATs are often individually underpowered due to the sample size being constrained by the host trial(s). A robust estimate of the effect of the SWAT intervention might therefore depend on the aggregation of replicated SWAT evaluations. It is not expected that a formal sample size calculation will always be done *The SWAT sample size depended on the host trial(s) [insert host trial name]; therefore no formal sample size calculation was performed, which is in line with SWAT methodology*. [insert any reasoning for a subsample of the host trial(s) being used – e.g. SWAT was included midway through the trial] State if interim analyses and/or stopping rules were planned or not If interim analyses and/or stopping rules were planned: *The following interim analyses were planned [state analyses here]. The stopping rules were [details here]*
**7b**	When applicable, explanation of any interim analyses and stopping rules for the SWAT	
**Randomisation: Sequence generation**		
**8a**	The method used to generate the random allocation sequence for the SWAT	Provide details of the method of randomisation: *Participants were randomised by [insert method with all methodological details]*
**8b**	Type of randomisation; details of any restriction (such as blocking and block size)	
**Allocation concealment mechanism**		
**9**	The mechanism used to implement the random allocation sequence (such as sequentially numbered containers), describing any steps taken to conceal the sequence until interventions were assigned for the SWAT	Provide details of the method of allocation concealment: *Allocation concealment was achieved by [insert method]*
**Implementation**		
**10**	Who generated the random allocation sequence, who enrolled participants, and who assigned participants to interventions for the SWAT	Provide details of randomisation sequence generation and implementation: *Randomisation was performed by [specify centre or personnel], [specify centre or personnel] enrolled participants and [specify centre or personnel] assigned the participant to the SWAT intervention or comparator*
**Blinding**		
**11a**	If done, who was blinded after assignment to the SWAT interventions (for example, participants, care providers, those assessing outcomes), and how	Explain who was blinded and if individuals were not blinded note the implications of this: *The [specify stakeholder group, e.g. participants, SWAT team members, outcome assessors, statisticians] were blind and the [specify stakeholder group, e.g. participants, SWAT team members, outcome assessors, statisticians] were not blind to the SWAT intervention.[Note implications of unblinded stakeholders as relevant]*
**11b**	If relevant, a description of the similarity of the SWAT interventions	
**Statistical methods**		
12a	Statistical methods used to compare groups for primary and secondary outcomes for the SWAT	All analyses for the SWAT should be preplanned, ideally detailed in a SWAT Statistical Analysis Plan (SAP), which might be a short component of the SWAT registry entry. Unless detailed thoroughly and extensively in a publicly available SWAT protocol, the analysis for each outcome should be detailed in the methods of the report. Alternatively, the SAP could be uploaded as supplementary material depending on the journal The analysis section should include the software used, the statistical methods (including significance level for hypothesis testing), and the population used for the analysis (e.g. intention-to-treat or per-protocol)
12b	Methods for additional analyses, such as subgroup analyses and adjusted analyses	
**Results**		
**Participant flow**		
13a	For each group, the numbers of participants who were randomly assigned, received intended SWAT intervention, and were analysed for the primary outcome of the SWAT	Provide a participant flow diagram that includes this data Include details of the host trial(s) participants excluded from the SWAT, with reasons, where appropriate
13b	For each group participating in the SWAT, losses, and exclusions after randomisation, together with reasons	
**Recruitment**		
14a	Dates defining the periods of recruitment and follow-up of the SWAT	Detail when SWAT activity took place: *Participant recruitment/follow-up took place between [insert dates]* If the SWAT ended or was stopped early: *The SWAT stopped [recruitment/follow up] early due to [insert text]*
14b	Why the SWAT ended or was stopped	
**Baseline data**		
**15**	A table showing baseline demographic and clinical characteristics for each group	The context of the host trial(s) for each SWAT evaluation is likely to be different and contextual information about the host trial(s) should be provided In addition to general information about the host trial(s) (see ‘Methods’), we suggest a table of participant baseline characteristics for those allocated to each group of the SWAT evaluation if these details are available. At a minimum, age, gender, and ethnicity should be reported per group in addition to any demographics deemed relevant by the host trial team, however, we encourage authors to refer to and report in accordance with PROGRESS-PLUS [27] where feasible
**Numbers analysed**		
16	For each group of the SWAT, the number of participants (denominator) included in each analysis and whether the analysis was by originally assigned groups	
**Outcomes and estimation**		
17a	For each primary and secondary outcome, results for each group, and the estimated effect size and its precision (such as 95% confidence interval)	Results should be presented in tables as far as possible rather than only being presented in the body of the text. To facilitate meta-analysis, SWATs should report the actual number of participants in each group in the SWAT evaluation A key element of SWAT evidence is their ability to be replicated. An important principle for reporting research is that new findings should be placed in the context of existing, relevant evidence. Therefore, we recommend, where possible, that an updated meta-analysis be included that presents the results of the current SWAT combined with previous evaluations of the SWAT intervention. Presentation as a cumulative meta-analysis is particularly helpful because it would help to inform judgements about the need for further evaluations of a SWAT intervention [7]
17b	For binary outcomes, the presentation of both absolute and relative effect sizes is recommended	
17c	Costs associated with the SWAT	Summarise the costs associated with the SWAT: *The total cost of the SWAT was [insert cost], which equates to [insert cost] per participant* Tabulate the additional costs to the trial incurred because of the SWAT, including total cost and cost per participant. This may include direct costs (e.g. printing, postage, animation) and indirect costs (e.g. staff time to prepare mailings). As SWAT evaluations generally need replication, it is useful for trialists to see the costs of both using the SWAT intervention *and* the cost of evaluating the SWAT should they wish to replicate the evaluation If a positive effect (irrespective of statistical significance) was identified, provide a cost per additional participant for whom there is a favourable result (e.g. cost per participant retained). Otherwise, note that cost per participant was not derived
**Ancillary analyses**		
18	Results of any other analyses performed on the SWAT data, including subgroup analyses and adjusted analyses, distinguishing pre-specified from exploratory	
**Harm**		
19	All important harm or unintended effects in each group that took part in the SWAT (for specific guidance, see CONSORT for harm)	If no harm or unintended effects were collected, this should also be noted
**Discussion**		
20	Interpretation consistent with results, balancing benefits and harm, and considering other relevant evidence	Within the discussion, reflect on the population demographics in the context of equality, diversity, and inclusion (e.g. Does the SWAT population reflect the host trial population(s)? If not, why not?)
**Limitations**		
21	SWAT limitations, addressing sources of potential bias, imprecision, and, if relevant, multiplicity of analyses for the SWAT	
**Generalisability**		
22	Generalisability (external validity, applicability) of the SWAT findings	
**Implications**		
**23**	Implications for trial practice and SWAT research	These could make use of the cumulative meta-analysis and Trial Forge Guidance 2 [7] on whether further evaluations of the intervention are warranted Consideration should be given to any other replications of the same SWAT and whether the findings are consistent with these or not. In addition, consideration should be given to the populations of other replications of the same SWAT when considering future SWAT research
**Other information**		
24	Registration Registration number and name of trial registry	Include the information for both the host trial(s) and SWAT It is recommended that SWATs are registered on a repository to ensure all SWATs performed can be included in the evidence base and support future replication The following repository is available to register SWATs: the Northern Ireland Methodology Hub’s SWAT repository (this repository is for SWATs and encourages replications of registered SWATs): https://www.qub.ac.uk/sites/TheNorthernIrelandNetworkforTrialsMethodology‌‌Research/SWATSWARInformation/Repositories/SWATStore/ SWATs may also be included in the ISRCTN trial registry (https://www.isrctn.com/) and/or the Clinical Trials database (https://clinicaltrials.gov/) as part of the host trial(s)
25	Protocol Where the full trial protocol can be accessed, if available	Include the information for both the host trial(s) and SWAT
26	Funding Sources of funding and other support (such as supply of drugs), role of funders	Include the information for both the host trial(s) and SWAT
**Additional**		
	Data sharing	We suggest authors make the data used to generate their results available as a supplementary file or through data-sharing platforms such as OSF (https://osf.io)

## Discussion

Our guideline draws on previous work by Madurasinghe et al. [14], adheres to the CONSORT 2010 guideline [15], and has been registered with the EQUATOR network. Throughout the development process, various stakeholders have been consulted, leading to iterative refinement of the guideline.

SWAT publications can be short and do not need to repeat information provided elsewhere (e.g. in the SWAT protocol on the SWAT repository at http://www.qub.ac.uk/sites/TheNorthernIrelandNetworkforTrialsMethodologyResearch/SWATSWARInformation/Repositories/SWATStore/). This guideline ought to make them an easy write and an easy read.

It is important to note that the guidance is currently designed for randomised studies embedded within a trial. Whilst this does not therefore cover the reporting of non-randomised SWATs, or randomised studies within cohorts for example, we anticipate these guidelines could easily be applied to these SWATs, albeit with some minor adaption, for example, non-reporting of intervention details and randomisation method. This corresponds with the approach Madurasinghe et. al*.* took with their earlier guideline [14].

SWATs play a key role in improving the evidence base for trial process decision-making, but they can only do so if their results are made publicly available promptly. If SWATs are published, ideally with an updated cumulative meta-analysis, this will provide more complete evidence on the effectiveness of alternative trial processes and will help trialists make better decisions.

## Conclusion

SWATs play a key role in improving the evidence base for trial process decision-making, but they can only do so if their results are made publicly available promptly. To ensure this, we need to remove the barriers to effective reporting of SWATs. The SWAT reporting guideline will aid authors, reviewers, and journal editors to produce and review clear, structured reports of randomised SWATs, whilst also adhering to the CONSORT guideline [15].

## Data Availability

Not applicable.
